# Longitudinal Insights into Sensorineural and Noise‐Induced Hearing Loss in Adolescents Aged 13‐18 Years

**DOI:** 10.1002/ohn.70042

**Published:** 2025-10-14

**Authors:** Stefanie N. H. Reijers, Jantien L. Vroegop, Danique E. Paping, Marieke Pronk, André Goedegebure, Bernd Kremer, Marc P. van der Schroeff

**Affiliations:** ^1^ Department of Otorhinolaryngology and Head and Neck Surgery Erasmus University Medical Center Rotterdam the Netherlands; ^2^ The Generation R Study Group Erasmus University Medical Center Rotterdam the Netherlands; ^3^ Dutch Consumer Safety Institute (VeiligheidNL) Amsterdam the Netherlands

**Keywords:** adolescents, longitudinal study, noise‐induced hearing loss, sensorineural hearing loss

## Abstract

**Objective:**

To describe the prevalence of sensorineural hearing loss (SNHL) and probable noise‐induced hearing loss (NIHL) among 18‐year‐olds, and to examine changes in prevalence and severity between ages 13 and 18.

**Study Design:**

Data were obtained from the Generation R Study, a prospective birth cohort in Rotterdam, the Netherlands.

**Setting:**

Audiometric assessments were conducted between 2016‐2019 (age 13) and 2020‐2024 (age 18).

**Methods:**

Main outcomes were SNHL and probable NIHL. SNHL was defined as a pure‐tone average >15 dB HL at low and/or high frequencies in one or both ears with a type A tympanogram. Probable NIHL was defined as a notch and/or high‐frequency hearing loss (HFHL), also with a type A tympanogram.

**Results:**

At age 18, 3347 adolescents were assessed. The prevalence of SNHL was 6.2%, and 12.9% met the criteria for probable NIHL. Among 2847 participants with data at both time points, prevalence rates remained stable over time. However, notches were more often bilateral at age 18, and HFHL thresholds had worsened significantly. Additionally, participants lost to follow‐up had relatively poorer hearing.

**Conclusion:**

At age 18, 6.2% of adolescents had SNHL, and 12.9% had signs of probable NIHL. While overall prevalence remained stable from 13 to 18 years, hearing deteriorated in severity, and selective drop‐out may have masked an increase. These findings emphasize the importance of continued monitoring and early prevention. Future research should explore the role of recreational noise exposure.

Hearing is essential for effective communication, safety, and psychosocial health throughout life. Early in life, it provides the foundation for critical developmental milestones, enabling children and adolescents to build communication skills, develop cognitive abilities, and perform academically.^1^, ^2^, ^3^, ^4^ However, even mild hearing loss—often untreated—can disrupt these processes, limiting social interactions and educational opportunities.^1^, ^5^, ^6^ While the prevalence of hearing loss increases with age, the underlying causes, such as excessive noise exposure, often begin in childhood.^7^, ^8^ Population‐based studies on hearing in children and adolescents are essential to inform public health measures such as systematic monitoring, early detection, and timely intervention. Despite their importance, such studies remain scarce. This study addresses this gap by examining the prevalence and longitudinal changes of sensorineural hearing loss (SNHL) and probable noise‐induced hearing loss (NIHL) in adolescents from a large Dutch cohort.

Hearing health can be conceptualized from a life course perspective—hearing loss develops gradually through cumulative damage from various non‐modifiable and modifiable risk factors.^9^ This perspective, endorsed by the World Health Organization, emphasizes proactive monitoring and early intervention to prevent progression. Recreational noise exposure is a major modifiable risk factor for NIHL, tinnitus, and hyperacusis,^10^ with evidence that it can cause irreversible damage.^11^, ^12^, ^13^ Adolescents are among the groups with the highest levels of exposure worldwide, which increases their risk of NIHL due to the cumulative effects of prolonged exposure to unsafe listening activities.^12^, ^14^ Listening to personal music players, visiting loud venues, and gaming are key sources of unsafe listening.^15^, ^16^, ^17^ Evidence suggests that the first 10 to 15 years of noise exposure are the most damaging.^18^ Early noise‐induced changes may also accelerate age‐related hearing loss.^19^, ^20^, ^21^

As adolescents navigate their formative years, early noise exposure can lead to a tipping point, worsening hearing health. Despite the importance of early monitoring, to our knowledge, only a few studies have used longitudinal data to describe SNHL and probable NIHL in young children or adolescents. Wang et al reported that the prevalence of SNHL (≥16 dB HL) in Australian children aged 11 to 12 years was 13.3%, highlighting the urgency for systematic monitoring.^22^ Data from the American NHANES study showed nearly 1 in 5 young adults (aged 20‐29) exhibited audiometric notches indicative of NIHL, largely due to prolonged noise exposure.^23^ Research by Paping et al. found that by age 13, 6.4% of Dutch children from the population‐based Generation R study experienced low‐ or high‐frequency SNHL (≥16 dB HL) in at least one ear, and 12.4% exhibited signs suggestive of NIHL (i.e., notches and/or high‐frequency hearing loss [HFHL]).^24^ This prevalence increased compared to data collected at age 9.^25^

This study builds on prior research by providing a longitudinal analysis of hearing thresholds in adolescents aged 13 and 18 years, using data from the Generation R study. It aims to describe the prevalence of and change in SNHL and probable NIHL, offering insights that could inform early public health preventive strategies.

## Methods

### Study Design and Subjects

This study used data from the Generation R study, a population‐based cohort in Rotterdam, the Netherlands, following children from fetal life into adulthood.^26^ Data collection includes questionnaires, interviews, and various measurements at the Erasmus Medical Center. For this study, data from ages 13 (2016‐2019) and 18 (2020‐2024) were analyzed. Hearing tests were carried out at the beginning of the test battery. Primary outcome measures were the prevalence of SNHL and probable NIHL. This study has two parts: Part I was cross‐sectional (18‐year‐olds), and Part II was longitudinal (ages 13 and 18). Ethical approval was obtained from the Medical Ethical Committee of Erasmus Medical Center Rotterdam, with written consent from participants.

### Pure Tone Audiometry

Pure‐tone audiometry was conducted in a soundproof booth adhering to ISO standard 8253‐1 for ambient sound pressure levels. Testing utilized a clinical audiometer (Decos audiology workstation; version 210.2.6 with AudioNigma interface) and TDH‐39P headphones with MX‐41/AR ear cushions, calibrated annually. Pure‐tone thresholds were assessed at frequencies of 0.5, 1, 2, 3, 4, 6, and 8 kHz. Thresholds were determined by the intensity level at which the tone was detected in 2 out of 3 ascents. Each ear was tested alternately, omitting bone conduction thresholds due to time constraints.

### Tympanometry

Middle ear function was assessed bilaterally unless contraindicated (e.g., excessive wax, otorrhea, acute otitis media, recent ear surgery). Tympanometry (Interacoustics AT235h, 226‐Hz probe tone) measured ear canal volume, middle ear pressure, and compliance. Ears with canal volume <0.3 mL were excluded to avoid collapse or wax obstruction. Tympanograms were classified per Jerger et al.: type A (normal; compliance ≥0.25 mL, pressure −100 to 100 daPa) and type B or C (external or middle ear pathology).^27^

#### Definition of Normal Hearing and Sensorineural Hearing Loss

The low‐frequency pure‐tone average (LPTA; 0.5, 1, 2 kHz) and high‐frequency pure‐tone average (HPTA; 3, 4, 6 kHz) were calculated from pure‐tone thresholds.^24^, ^25^, ^28^, ^29^ Normal hearing was defined as LPTA and HPTA ≤ 15 dB in both ears. Hearing levels were classified as slight (16‐25 dB HL), mild (26‐40 dB HL), moderate (41‐55 dB HL), moderately severe (56‐70 dB HL), severe (71‐90 dB HL), or profound (≥91 dB HL).^30^ Hearing loss (>15 dB HL) with a type A tympanogram was classified as SNHL; with type B or C as probable conductive hearing loss (CHL), although mixed or underlying SNHL could not be excluded.

#### Definition of Probable Noise‐Induced Hearing Loss

As described in previous Generation R studies,^24^, ^25^ audiometric indicators suggestive of NIHL included the presence of a notch and/or HFHL in one or both ears, combined with a type A tympanogram. A notch was defined according to the criteria of Niskar et al., which include: (1) thresholds of 15 dB HL or less at 0.5 and 1 kHz; (2) the poorest threshold at 3, 4, or 6 kHz being at least 15 dB or worse than the poorest threshold at 0.5 and 1 kHz; and (3) a threshold at 8 kHz that is at least 10 dB better than the poorest threshold at 3, 4, or 6 kHz.^31^ HFHL was defined as (1) hearing thresholds of 15 dB HL or less at 0.5 and 1 kHz, and (2) an average threshold at 3, 4, 6, and 8 kHz greater than 15 dB HL.

### Statistical Analysis

Analyses were performed in SPSS version 24. Hearing thresholds were compared using the paired samples *t*‐test or, when not normally distributed, the Wilcoxon signed‐rank test. Prevalence rates of hearing loss were calculated with 95% confidence intervals (CIs). Differences in proportions were evaluated using McNemar's test. An attrition analysis assessed whether the prevalence of SNHL and probable NIHL differed between participants excluded due to missing data and those in the longitudinal sample. Statistical significance was set at *P* < .05.

## Results

### Part I—Cross‐Sectional Analysis

A total of 3630 adolescents visited the research center between October 2020 and June 2024, of whom 3491 (96.2%) completed pure‐tone audiometry at all frequencies in both ears. Of these, 144 (4.1%) had a probable CHL or hearing loss of unknown origin in at least 1 ear, based on tympanometry, and were excluded. The remaining 3347 participants had a mean age of 18 years and 5 months (SD 8 months; range 16 years and 2 months–21 years and 9 months), and 1778 (53.1%) were girls. Demographic characteristics are shown in Table 1. The mean (SD) LPTA for the total cohort was 4.8 (4.6) dB HL for right ears and 5.1 (5.4) dB HL for left ears. For HPTA, the mean (SD) was 6.3 (5.6) dB HL for right ears and 7.1 (6.4) dB HL for left ears. Mean thresholds stratified by hearing status (normal hearing vs SNHL) are shown in Table 2.

**Table 1:** Demographic Characteristics of the Participants Included in the Cross‐Sectional Analysis at Age 18 (n = 3347)

Characteristics	Included (n = 3347)	SNHL (n = 209)	NIHL (n = 433)
Age, mean (SD), y	18 year, 5 mo (0.7)	18 year, 5 mo (0.7)	18 year, 4 mo (0.6)
Gender, No. (%)			
Female	1778 (53.1)	96 (45.9)	216 (49.9)
Male	1565 (46.8)	112 (53.6)	217 (50.1)
X	4 (0.1)	1 (0.5)	0
Ethnicity, No. (%)			
Western	2399 (71.7)	149 (71.3)	314 (72.5)
Non‐Western	878 (26.2)	56 (26.8)	113 (26.1)
Missing	70 (2.1)	4 (1.9)	6 (1.4)
Educational level participant, No. (%)			
Secondary education, phase 1	43 (1.3)	3 (1.4)	6 (1.4)
Secondary education, phase 2	1239 (37.0)	68 (32.5)	153 (35.3)
Higher education, phase 1	355 (10.6)	21 (10.1)	46 (10.6)
Higher education, phase 2	420 (12.6)	27 (12.9)	59 (13.6)
Missing	1290 (38.5)	90 (43.1)	169 (39.0)
Educational level mother, No. (%)			
Lower	127 (3.8)	14 (6.7)	16 (3.7)
Intermediate	1206 (36.0)	81 (38.8)	153 (35.3)
Higher	1929 (57.6)	109 (52.2)	258 (59.6)
Missing	85 (2.5)	5 (2.4)	6 (1.4)
Household income, No. (%)			
<€2800	652 (19.5)	56 (26.8)	118 (27.3)
≥€2800	1892 (56.5)	122 (58.4)	260 (60.1)
Missing	803 (24.0)	31 (14.8)	55 (12.7)

Abbreviations: Higher education, phase 1, Higher professional education; Higher education, phase 2, University or scientific education; Secondary education, phase 1, Special secondary education, preliminary vocational education, or lower vocational/general secondary education; Secondary education, phase 2, General secondary education or senior secondary vocational education; X, individuals identifying outside the binary categories of female and male, including nonbinary, genderqueer, or other gender‐diverse identities.

**Table 2:** Hearing Thresholds at Both Time Points

	Baseline (13 years)		Follow‐up (18 years)	
	Right	Left	Right	Left
Total cohort				
LPTA (dB HL), mean (SD)	5.1 (4.1)	5.0 (4.3)	4.8 (4.6)	5.1 (5.4)
HPTA (dB HL), mean (SD)	6.2 (4.8)	6.6 (4.9)	6.3 (5.6)	7.1 (6.4)
Normal hearing				
LPTA (dB HL), mean (SD)	4.9 (3.6)	4.8 (3.8)	4.2 (3.5)	4.3 (3.9)
HPTA (dB HL), mean (SD)	5.8 (3.8)	6.2 (4.0)	5.3 (4.0)	5.8 (4.2)
Sensorineural hearing loss				
LPTA (dB HL), mean (SD)	21.9 (8.8)	21.0 (7.1)	19.6 (7.9)	23.0 (9.1)
HPTA (dB HL), mean (SD)	22.2 (8.2)	21.3 (8.4)	22.1 (7.4)	22.4 (8.6)

The low‐frequency pure‐tone averages (LPTAs) and high‐frequency pure‐tone averages (HPTAs) for both right and left ears at baseline (13 years) and during follow‐up (18 years). Results are shown for the entire cohort, as well as for normal hearing and sensorineural hearing loss.

### Sensorineural Hearing Loss

A total of 209 (6.2%) (95% confidence interval [CI], 5.4%‐7.1%) participants were classified as SNHL. Most cases were unilateral (n = 168, 80.4%); 56 (33.3%) in the right ear, and 112 (66.7%) in the left ear. Among the 209 participants with SNHL, the degree was slight (16‐25 dB HL) in 81.3%, mild (26‐40 dB HL) in 15.8%, moderate (41‐55 dB HL) in 1.4%, and moderately severe or severe (≥56 dB HL) in 1.4%.

### Probable Noise‐Induced Hearing Loss

Of the 3347 participants included in the analyses, 294 (8.8%) (95% CI, 7.9%‐9.6%) participants met the criteria for a notch, 198 (5.9%) (95% CI, 5.1%‐6.7%) for HFHL, and 59 (1.8%) (95% CI, 1.3%‐2.2%) for both a notch and HFHL, in one or both ears. Overall, 433 (12.9%) (95% CI, 11.8%‐14.1%) participants met the criteria of probable NIHL (notch and/or HFHL) (Table 3). Among the 294 participants with a notch, 256 (87.1%) were unilateral (32.8% in the right ear, and 67.2% in the left ear). In the majority of the participants with a notch (92.9%), the notch was present at a single frequency, mostly at 6 kHz. In 6.1% of the cases, two frequencies were involved, and in 1.0% of these participants, three frequencies were involved. Of the 198 participants with HFHL, the majority was unilateral (n = 166, 83.8%). Most of these participants had HFHL of slight degree (n = 163, 82.3%), 16.2% (n = 32) of mild degree, and 1.5% (n = 3) of moderate to profound degree.

**Table 3:** Participants With Probable NIHL (Notch and/or HFHL) in the Cross‐Sectional Analysis at Age 18 (n = 3347)

Category	No. of participants (%)
Notch	
Notch only	235 (7.0)
Notch total	294 (8.8)
HFHL	
HFHL only	139 (4.2)
HFHL total	198 (5.9)
Both a notch and HFHL	59 (1.8)
**Total probable NIHL**	433 (12.9)

In this table, “notch only” refers to participants with a notch but no HFHL, and “HFHL only” to those with HFHL but no notch. The totals (“notch total” and “HFHL total”) also include participants with both a notch and HFHL. The category “both a notch and HFHL” refers to cases where both features were present in the same ear. “Total probable NIHL” includes all participants with a notch and/or HFHL in at least 1 ear.

### Part II—Longitudinal Analysis

A total of 3044 participants completed pure‐tone audiometry at time points 13 and 18 years, of whom 197 (6.5%) were excluded due to the presence of probable conductive or unclassified hearing loss at one or both visits. This resulted in the inclusion of 2847 participants with repeated measurements. Of these participants, 53.2% were girls. The mean interval between 13 years and follow‐up at 18 years was 4 years and 10 months (SD 8 months).

There were only minor, nonsignificant changes in the prevalence of SNHL, notches, HFHL, and probable NIHL between ages 13 and 18. SNHL prevalence was 5.3% (95% CI: 4.3%‐6.5%) at age 13 and 5.2% (95% CI: 4.2%‐6.4%) at age 18. The prevalence of notches increased slightly from 7.9% (95% CI: 6.8%‐9.2%) to 8.4% (95% CI: 7.3%‐9.7%). HFHL was observed in 5.2% (95% CI: 4.2%‐6.4%) at age 13 and 5.1% (95% CI: 4.1%‐6.3%) at age 18. Probable NIHL remained relatively stable, with a prevalence of 11.7% (95% CI: 10.7%‐12.8%) and 11.8% (95% CI: 10.8%‐12.8%), respectively.

**Figure 1:**
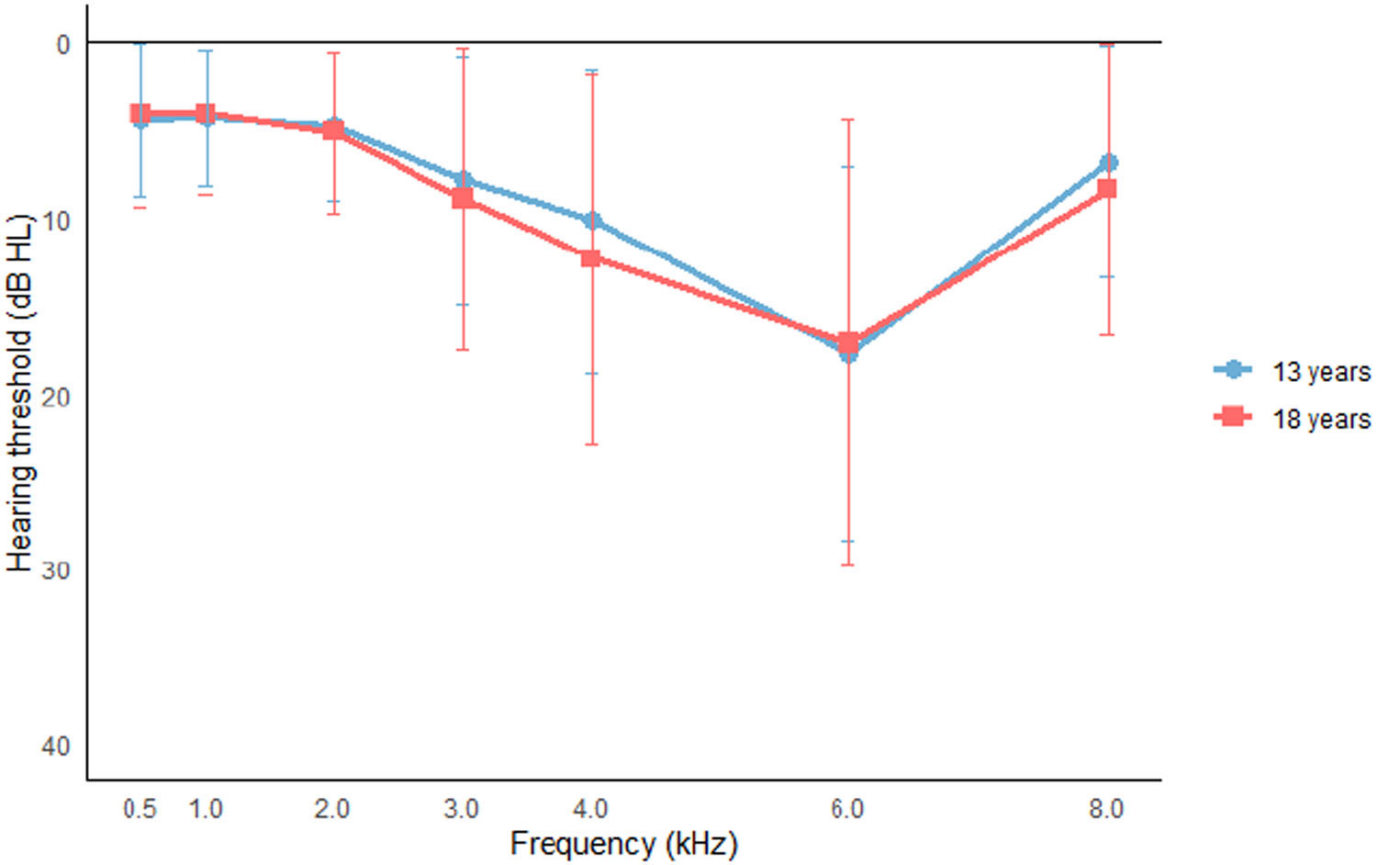
Hearing thresholds of participants with a notch. Mean thresholds of participants with a notch at 13 (n = 225) and 18 years (n = 238); poorest ear shown if bilateral. Error bars indicate ± 1 SD.

**Figure 2:**
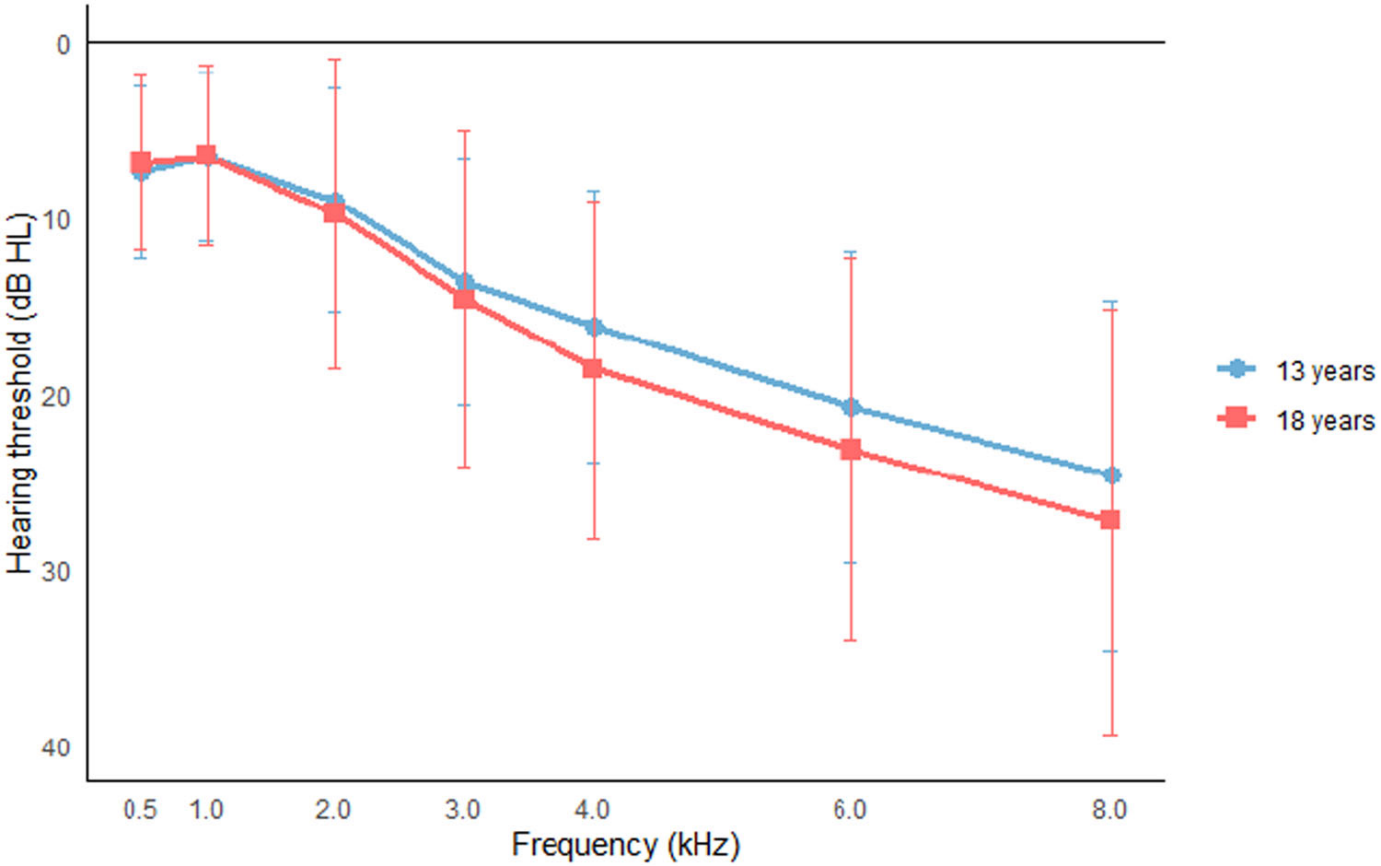
Hearing thresholds of participants with HFHL. Mean thresholds of participants with HFHL at 13 (n = 148) and 18 years (n = 144); poorest ear shown if bilateral. Error bars indicate ± 1 SD.

Within the group of participants with a notch at 13 years (n = 225), 20.9% had a notch of mild degree or worse (≥26 dB HL), compared to 21.4% during follow‐up at 18 years (*P* = .581). The proportion of participants with a bilateral notch increased significantly from 7.1% to 12.2% during follow‐up (*P* < .001). Among the participants with HFHL at baseline, 8.1% had HFHL of mild degree or worse (≥26 dB HL), compared to 13.9% during follow‐up, indicating a significant increase in degree of hearing loss (*Z* = −8.017, *P* < .001). The proportion of participants with a bilateral HFHL was 14.2% at baseline and 14.6% during follow‐up. This difference was not significant. Figures 1 and 2 visualize the mean hearing thresholds for the notches and HFHL, while Table 4 shows the distribution of the degrees of hearing loss.

**Table 4:** Distribution of the Degree of a Notch and HFHL at Baseline (13 Years) and During Follow‐Up (18 Years) in Participants With Repeated Measurements (n = 2847)

	Notch at 13 years	Notch at 18 years	HFHL at 13 years	HFHL at 18 years
	(n = 225)	(n = 238)	(n = 148)	(n = 144)
A notch within the range of normal hearing, n (%)	36 (16.0)	53 (22.3)	0	0
Slight hearing loss (16‐25 dB HL), n (%)	142 (63.1)	134 (56.3)	136 (91.9)	124 (86.1)
Mild hearing loss (26‐40 dB HL), n (%)	42 (18.7)	46 (19.3)	10 (6.8)	18 (12.5)
Moderate hearing loss (41‐55 dB HL), n (%)	5 (2.2)	5 (2.1)	2 (1.4)	2 (1.4)
Moderately severe (56‐70 dB HL), severe (71‐90 dB HL) to profound hearing loss (>90), n (%)	0	0	0	0

Degree of hearing loss was classified according to the American Speech‐Language‐Hearing Association guidelines. In case of bilateral hearing loss, the degree of the poorest thresholds is presented.

The attrition analysis showed that in the group of participants that completed pure‐tone audiometry at age 13 but were excluded from the longitudinal analysis due to missing data at age 18 (n = 1637, 57.5% of the longitudinal sample), a significant difference in the prevalence of SNHL was found at age 13 (7.0% in the excluded group vs 5.3% in the longitudinal sample, *P* = .023). In contrast, no significant difference was observed for probable NIHL (12.9% in the excluded group vs 11.7% in the longitudinal sample, *P* = .230). Among participants with audiometric data available only at age 18 (n = 420, 14.8% of the longitudinal sample), a significant difference in the prevalence of SNHL was also observed (9.3% in the excluded group vs 5.2% in the longitudinal sample, *P* < .001). Additionally, a significant difference in the prevalence of probable NIHL was found (16.4% in the excluded group vs 11.8% in the longitudinal sample, *P* = .007).

## Discussion

This study provides longitudinal insights into the prevalence and changes in SNHL and probable NIHL among adolescents aged 13 to 18. At age 18, cross‐sectional analysis showed a prevalence of 6.2% for SNHL and 12.9% for probable NIHL. Longitudinal analysis revealed stable rates over time, but notches were more common at age 18 than at 13, and HFHL thresholds worsened at age 18 among those with existing HFHL at 13. This suggests that some adolescents may be more vulnerable to continued deterioration, even when overall prevalence remains stable. This pattern may reflect individual differences in susceptibility to noise‐induced damage, as described by Hong et al.^32^ Prevalence rates may have been slightly underestimated due to selective dropout, as attrition analysis showed significantly higher SNHL rates in both excluded groups (7.0% vs 5.3%; 9.3% vs 5.2%) and higher probable NIHL rates in one excluded group (16.4% vs 11.8%).

Taking a broader perspective, our findings on probable NIHL prevalence were partly consistent with other studies.^24^, ^31^ For example, Niskar et al. defined NIHL solely based on the presence of a notch and reported an increase from 8.5% in American children aged 6 to 11 to 15.5% in adolescents aged 12 to 19, using NHANES data.^31^ In contrast, the notch prevalences we and Paping et al. observed in the Generation R sample were lower. We found 8.8% with a notch at age 18 (cross‐sectional), and 7.9% (age 13) and 8.4% (age 18) in the longitudinal analysis, with no significant increase. Paping et al. reported a cross‐sectional prevalence of 8.0% at age 13, and longitudinal rates of 4.3% at age 9 and 7.8% at age 13, with a significant increase in probable NIHL between ages 9 and 13 (9.8% vs 11.7%).^24^ Thus, while Generation R data showed lower NIHL prevalences than Niskar, there was a significant increase between ages 9 and 13, mostly due to more children with a notch,^24^ and increased HFHL and bilateral notches between 13 and 18 (this study). Given the apparent plateau in prevalence from 13 to 18, it is unclear why no further increase occurred. One explanation is that most damage occurred before age 13, with limited deterioration later. Alternatively, greater awareness among adolescents and event organizers may have reduced harmful exposure. Supporting this, children with a notch but no HFHL did not show deterioration over time, unlike those with existing HFHL, suggesting a notch alone does not necessarily predict progression to SNHL.

Looking at SNHL, the 6.2% prevalence in our study is lower than the 11.2% reported by Humes et al. for American youth aged 6 to 19 years,^33^ and also lower than the 13.3% reported by Wang for Australian 11‐to‐12‐year‐old children.^22^ Direct comparison with Humes et al. however is difficult, as they included both SNHL and CHL in their definition, which led to a higher overall prevalence.^33^ In our cohort, unilateral SNHL was notably more often observed in the left ear (66.7%). This contrasts with findings from other pediatric cohorts, which typically report an approximately equal distribution between ears. For example, a study of 145 children with unilateral SNHL found 51.8% of cases in the left ear.^34^ The reason for this asymmetry is unclear, but potential explanations include random variation, test procedures, unrecognized environmental factors, structural abnormalities, or a combination of these factors. Imaging studies could shed a light on the variance explained by structural abnormalities. A recent meta‐analysis found that imaging revealed potentially causative abnormalities in approximately 35% to 37% of children with unexplained unilateral SNHL.^35^ It is important to note that this meta‐analysis mainly included children with more severe degrees of unilateral hearing loss, whereas in our study, most cases involve slight to mild unilateral hearing loss. These anomalies are considered nonmodifiable risk factors and may contribute to the development and persistence of hearing loss in this population.

In summary, variation in prevalence across studies may stem from differences in definitions of SNHL and probable NIHL, but also from other methodological variations, including covered age ranges, sample diversity, and cultural and environmental factors affecting hearing health.

Lastly, NIHL definitions should ideally be supported by detailed information on participants' past noise exposure, but including such reliable measures in general cohort studies remains a major methodological challenge.^36^ This may partly explain the mixed results in previous studies.^37^, ^38^, ^39^ Although experts agree that high levels and durations of recreational noise exposure can cause permanent auditory damage, specific unsafe thresholds and associated risks remain unclear.^36^, ^40^ Future studies should assess various noise sources (e.g., personal music players, gaming, nightlife) to estimate total recreational exposure and its cumulative impact on hearing and NIHL prevalence. Additionally, it would be valuable to examine whether bilateral notches—especially in individuals initially presenting with unilateral notching—are associated with a higher risk of HFHL. This could clarify whether a notch reflects early‐stage progressive cochlear damage. Other factors such as genetics, pre‐existing conditions, and ototoxic environmental exposures may also contribute to SNHL and affect NIHL rates, but are often unaccounted for. In summary, long‐term cohort data are needed to capture how cumulative noise exposure and other risk factors manifest in the audiogram over time, to guide prevention.

### Strengths and Limitations

This study's strengths include its prospective design, large sample size, and standardized hearing assessments by trained staff. Limitations are the absence of otoscopy and bone‐conduction audiometry due to time constraints. Congenital hearing loss was not considered due to lack of data. Lastly, the definition of probable NIHL has some uncertainty, as lifetime noise exposure was not assessed.

## Conclusion

In this prospective cohort study, 6.2% of 18‐year‐olds had SNHL, and 12.9% showed a notch and/or HFHL suggesting probable NIHL. Although the prevalence rates remained stable from ages 13 to 18, bilateral notches and HFHL severity increased. These results emphasize the importance of ongoing monitoring of hearing health in adolescents. Further studies should focus on examining datasets from longitudinal adolescent cohorts covering long follow‐ups, and should examine associations with noise exposure.

## Author Contributions

**Stefanie N. H. Reijers**, study design, data acquisition, data analysis, drafting and revising the manuscript; **Jantien L. Vroegop**, supervision, interpretation of findings, critical revision of the manuscript; **Danique E. Paping**, data interpretation, critical revision of the manuscript; **Marieke Pronk**, interpretation of findings, critical revision of the manuscript; **André Goedegebure**, interpretation of audiological data, critical revision of the manuscript; **Bernd Kremer**, supervision, critical revision of the manuscript; **Marc P. van der Schroeff**, conceptualization, supervision, interpretation of findings, critical revision of the manuscript.

## Disclosures

### Competing interests

None.

### Funding source

Netherlands Organization for Health Research and Development; Ministry of Health, Welfare and Sport; Netherlands Organization for Scientific Research (NWO); Erasmus University Rotterdam; Erasmus Medical Center, Rotterdam.
